# PRO-C3-Levels in Patients with HIV/HCV-Co-Infection Reflect Fibrosis Stage and Degree of Portal Hypertension

**DOI:** 10.1371/journal.pone.0108544

**Published:** 2014-09-29

**Authors:** Christian Jansen, Diana J. Leeming, Mattias Mandorfer, Inger Byrjalsen, Robert Schierwagen, Philipp Schwabl, Morten A. Karsdal, Evrim Anadol, Christian P. Strassburg, Jürgen Rockstroh, Markus Peck-Radosavljevic, Søren Møller, Flemming Bendtsen, Aleksander Krag, Thomas Reiberger, Jonel Trebicka

**Affiliations:** 1 Department of Internal Medicine I, University of Bonn, Bonn, Germany; 2 Nordic Bioscience, Fibrosis Biology and Biomarkers, Herlev, Denmark; 3 Division of Gastroenterology and Hepatology, Department of Internal Medicine III, Medical University of Vienna, Vienna, Austria; 4 German Centre for Infection Research (DZIF), partner site Bonn-Cologne, Bonn, Germany; 5 Center of Functional and Diagnostic Imaging and Research, Department of Clinical Physiology, Hvidovre Hospital, University of Copenhagen, Copenhagen, Denmark; 6 Gastro Unit, Medical Division, Hvidovre Hospital, Faculty of Health Sciences, University of Copenhagen, Copenhagen, Denmark; 7 Department of Medical Gastroenterology, Odense University Hospital, Odense, Denmark; University of Navarra School of Medicine and Center for Applied Medical Research (CIMA), Spain

## Abstract

**Background:**

Liver-related deaths represent the leading cause of mortality among patients with HIV/HCV-co-infection, and are mainly related to complications of fibrosis and portal hypertension. In this study, we aimed to evaluate the structural changes by the assessment of extracellular matrix (ECM) derived degradation fragments in peripheral blood as biomarkers for fibrosis and portal hypertension in patients with HIV/HCV co-infection.

**Methods:**

Fifty-eight patients (67% male, mean age: 36.5 years) with HIV/HCV-co-infection were included in the study. Hepatic venous pressure gradient (HVPG) was measured in forty-three patients. The fibrosis stage was determined using FIB4 -Score. ECM degraded products in peripheral blood were measured using specific ELISAs (C4M, MMP-2/9 degraded type IV collagen; C5M, MMP-2/9 degraded type V collagen; PRO-C3, MMP degraded n-terminal propeptide of type III collagen).

**Results:**

As expected, HVPG showed strong and significant correlations with FIB4-index (r_s_ = 0.628; p = 7*10^−7^). Interestingly, PRO-C3 significantly correlated with HVPG (r_s_ = 0.354; p = 0.02), alanine aminotransferase (r_s_ = 0.30; p = 0.038), as well as with FIB4-index (r_s_ = 0.3230; p = 0.035). C4M and C5M levels were higher in patients with portal hypertension (HVPG>5 mmHg).

**Conclusion:**

PRO-C3 levels reflect liver injury, stage of liver fibrosis and degree of portal hypertension in HIV/HCV-co-infected patients. Furthermore, C4M and C5M were associated with increased portal pressure. Circulating markers of hepatic ECM remodeling might be helpful in the diagnosis and management of liver disease and portal hypertension in patients with HIV/HCV coinfection.

## Introduction

Combined antiretroviral treatment (cART) is able to control the HIV replication in the majority of HIV patients, and thereby reduce mortality [1], [2]. While a decrease of AIDS-related deaths are noted, mortality related to chronic liver diseases is steadily increasing in HIV patients [3]–[5]. Nearly 24% of non-AIDS related deaths in HIV-infected patients are due to end-stage liver disease (ESLD), with 66% being attributed to HCV co-infection and 17% to HBV co-infection [5]. This represents an important socioeconomic problem, given the fact that more than 30% of HIV-patients in Europe and USA are co-infected [4]. HCV replication is increased in HIV coinfection [6], whereas HCV-specific immune responses are attenuated [7]–[10], which may be the cousal factors to the progression of liver fibrosis and accelerated development of ESLD in patients with HCV/HIV co-infection as compared to HCV mono-infected patients [11], [12]. ESLD is associated with complications and mortality mainly due to portal hypertension. Measurement of hepatic venous pressure gradient (HVPG) is the best available tool to directly assess portal hypertension[13]–[16]. Thus, the early detection of fibrosis and portal hypertension especially in HIV/HCV co-infected patients represents an unmet clinical need.

During progression of liver fibrosis both, the synthesis and degradation of extracellular matrix (ECM) are increased – referred to as ECM remodeling [17]–[19]. Endopeptidases, such as matrix metalloproteinases (MMPs) MMP-2 and MMP-9 are enhanced during ECM remodeling, especially in HIV-positive patients [20]–[22]. During the synthesis and degradation of collagen by MMPs small fragments are released into the circulation, and their levels mirror the extent of liver dysfunction and portal hypertension in patients with alcoholic cirrhosis [17].

The aims of the present study were to evaluate circulating collagen fragments as biomarkers for (i) liver fibrosis stage and (ii) portal hypertension in patients with HIV/HCV coinfection.

## Materials and Methods

### Study design

We retrospectively included fifty-eight (67% male) patients with HIV/HCV coinfection. The median age was 36.5 years (range 19 to 63). HVPG was measured in forty-three patients. The fibrosis stage was determined using Fibroscan and FIB4-Score (Table 1). Circulating collagen fragments in peripheral serum were measured in all patients during routine blood sampling as previously described (Table S1). Furthermore, biochemical parameters were analyzed using standard methods (Table 2). The patients gave their written consent for the procedures and the local ethics committee of the University of Bonn (Nr. 069/10) as well as the local ethics committee of the University of Vienna (EK 005/2005) approved the study [23] in accordance with the Declaration of Helsinki.

**Table 1:** Clinical parameters of patients.

Parameters	n	value
Gender (female/male, % male)	58	19/39 (67%)
Age	58	36.5 (19–63)
Weight (kg)	58	69 (45–103)
HVPG (mmHg)	43	3 (2–13)
Liver Stiffness (kPa)	46	7 (3–22)
FIB-4 Index	43	2 (0–4)

HVPG: hepatic venous pressure gradient; FIB-4 Index: Fibrosis 4 Score.

Data are shown as median (range) and n numbers of patients per group.

**Table 2:** Biochemical parameters of included patients.

Parameters	n	value
ALT (U/L)	48	71 (13–348)
PLT (10^9^/L)	48	184 (55–330)
HCV-RNA (IU/mL)	48	1,052,500 (5,590–36,700,000)
CD4 (U/µL)	48	515 (134–1,222)
PRO-C3 (ng/mL)	58	22 (10–50)
C4M (ng/mL)	58	141 (54–411)
C5M (ng/mL)	58	375 (182–727)

ALT, alanine aminotransferase; PLT, platelets; HCV-RNA, hepatitis C virus ribonucleic acid; CD4, CD4+ T helper cells; PRO-C3, degraded n-terminal propeptide of type III collagen; C4M, degraded type IV collagen; C5M, degraded type V collagen. Data are shown as median (range).

### Measurement of HVPG, liver stiffness by transient elastography and calculation of FIB-4 Index

HVPG measurements were performed as previously described [13], [24], [25]. Under local anesthesia and ultrasound guidance, the right internal jugular was canulated by usage of the Seldinger technique. A balloon catheter was placed under continuous radiological control in a major hepatic vein. To calculate HVPG, three repeated measurements of free and wedged hepatic venous pressure were performed. Pressure curves were continuously recorded using a licensed software (S5 Collect, Vienna, Austria).

Transient elastography (Fibroscan, Echosens, France) was used for measurement liver stiffness as previously described [25]–[27].

FIB4-index was calculated in 43 patients, as previously described [28]. FIB4-index is based on AST, ALT, platelet count, and age: FIB4 = (age [years] × AST [IU L^−1^])/(platelet count [10^9^ L^−1^]×√ALT [IU L^−1^]).

### Enzyme-linked immunosorbentassay

Specific enzyme-linked immunosorbentassay (ELISA) were assessed in the described patient group following a protocol as previously described [17] to detect degradation of ECM fragments. In detail: PRO-C3 (true collagen type III formation) [29] C4M (MMP-2/9 degraded type IV collagen) [30] and C5M (MMP-2/9 degraded type V collagen) [31] were quantified (Table S1).

### Statistical analysis

Mann-Whitney-U test was used for unpaired comparisons. The correlations were analyzed by the use of Spearman correlation coefficient. Significant differences in levels of circulating biomarkers in peripheral blood between more than two groups were analyzed using Kruskal-Wallis-Test. p<0.05 was considered as statistically significant. Statistical analyses were performed with SPSS 22 (SPSS Inc. Chicago, IL, USA).

## Results

### Clinical, biochemical, and haemodynamic characteristics of patients

The clinical characteristics of the study population are shown in Table 1. The biochemical parameters including the levels of ECM markers are shown in Table 2. All patients were treated with cART and HIV viral load was under detection level.

**Figure 1:**
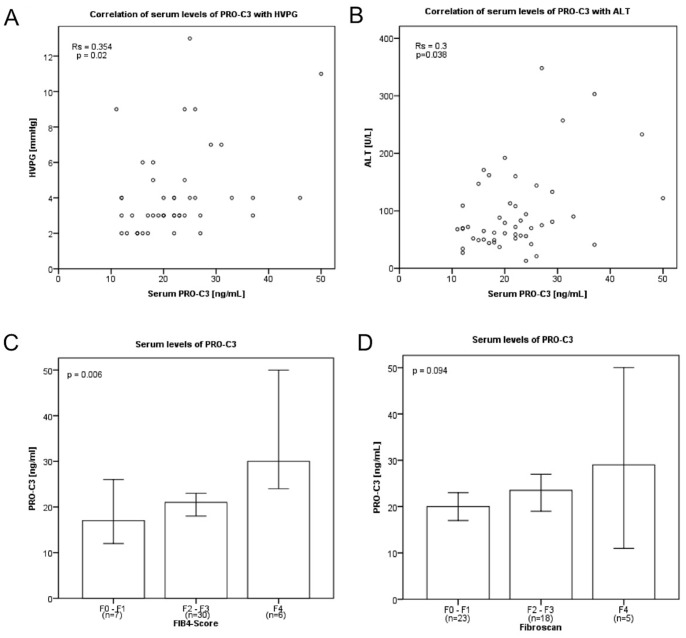
Levels of PRO-C3 measured in blood of patients with HIV/HCV co-infection correlated with hemodynamic and liver function parameters as well with FIB4-Score. The levels of PRO-C3 (A) correlated with HVPG (r_s_ = 0.354; p = 0.02) as well as the levels of ALT (r_s_ = 0.3; p = 0.038) (B). Furthermore significant higher levels of PRO-C3 could be observed in patients with advanced fibrosis stratified using FIB4-Score (p = 0.006) (C). Instead using fibroscan to stratify patient in the same way only a tendency could be seen (p = 0.094) (D).

FIB-4 index was calculated in forty-three patients as previously described [28]. Seven patients showed no or F1 fibrosis, 30 patients showed stage F2 or F3 and six patients showed fibrosis F4 (Table 1; Figure 1C). Using transient elastography with a median of 7 kPa (range 3–22 kPa), more patients showed a mild fibrosis (F0/1 n = 23; F2/3 n = 18; F4 n = 5) (Table 1). HVPG was measured in forty-three patients. The median pressure was 3 mmHg (range: 2–13 mmHg). HVPG correlated with FIB-4 index **(**r_s_ = 0.628; p = 7*10^−7^), liver stiffness (r_s_ = 0.621; p = 9*10^−5^), and inversely with platelet counts (r_s_ = −0.366; p = 0.016) (Figure S1). The strong correlation of HVPG with liver stiffness serves as internal quality control of the data raised in the patients.

### Relationship between PRO-C3 levels and parameters of liver injury

Interestingly, serum PRO-C3 levels significantly correlated with HVPG (r_s_ = 0.354; p = 0.020; Figure-1A; Table 3). Furthermore, PRO-C3 levels significantly correlated with ALT as a marker of liver injury (r_s_ = 0.30; p = 0.038; Figure 1B; Table 3).

**Table 3:** Correlations of PRO-C3 with fibrosis, portal hypertension and liver function.

PRO-C3	n	Rs	p-value
FIB4-Score	43	0.323	0.035
HVPG (mmHg)	43	0.354	0.020
ALT (U/L)	48	0.300	0.038

ALT, alanine aminotransferase; HVPG hepatic venous pressure gradient.

Moreover, serum PRO-C3 correlated with FIB-4 index (r_s_ = 0.323; p = 0.035; Table 3) and was significantly higher in patients with advanced fibrosis as indicated either by FIB-4 index or transient elastography (Figure 1C, 1D).

### Relationship between serum C4M and C5M levels and parameters of liver injury

**Figure 2:**
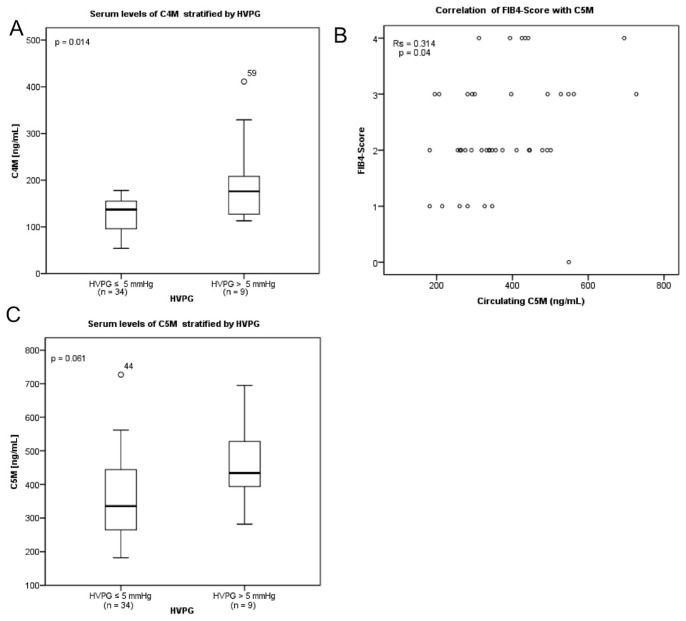
FIB4 score correlated significant with C5M (B). Serum levels of C5M (C) and C4M (A) stratified between patients with higher and lower or equal hepatic venous pressure gradient than 5 mmHg differ. Levels of serum C5M (B) correlated significantly with FIB4-score (r_s_ = 0.314; p = 0.04). Stratified patients between higher and lower hepatic venous pressure gradient than 5 mmHg showed significant differ in levels of serum C4M (p = 0.014) (A). Levels of C5M (p = 0.061) (C) only showed a tendency. Data are shown by using box-plots and were analyzed with the Wilcoxon test.

Levels of serum C5M significantly correlated with FIB-4 index (r_s_ = 0.314; p = 0.04; Figure 2B) and were higher in patients with portal hypertension defined as HVPG above 5 mmHg (Figure 2C). Interestingly, serum levels of C4M were significant higher (p = 0.014) in patients with portal hypertension (Figure 2A).

## Discussion

This study demonstrated that serum PRO-C3 levels may serve as biomarkers for the extent of liver fibrosis, liver injury, and portal hypertension in HIV/HCV-co-infected patients. Additional markers of ECM remodeling such as serum C4M and C5M levels might be useful in these patients to non-invasively diagnose the presence of portal hypertension.

ECM remodelling occurs as an attempt to repair damaged tissue response to liver injury [14], [32]–[34], while ongoing hepatic injury leads to a non-regenerative wound healing characterized by high turnover of ECM and ultimately, a net accumulation of fibrotic scar tissue in the liver. The deposition of fibrotic material continuously increases intrahepatic vascular resistance to portal blood flow and thereby causing portal hypertension [35], [36]. Therefore, the progression of fibrosis and development of portal hypertension are closely associated [37]. Especially in patients with HIV/HCV co-infection the evaluation of fibrosis and portal hypertension represents a major issue, since disease progression is accelerated [38] and liver-related mortality is increasing [3]–[5]. These patients might be asymptomatic until clinical decompensation occurs, but at this stage preventing morbidity and mortality represents a major clinical challenge. Therefore, non-invasive biomarkers are needed for early detection of liver fibrosis and portal hypertension - before clinically significant complications and decompensation occur.

The present study showed that the collagen type III formation marker PRO-C3 correlated with liver injury, fibrosis, and portal hypertension in HIV/HCV co-infected patients. This marker was previously described to derive from the diseased liver and reflects fibrosis and portal hypertension in animal models of fibrosis [18] and in humans with alcoholic cirrhosis [17]. The PRO-C3 ELISA is different and may be superior to other commercial available assays since other commercial PIIINP assays available utilize either a monoclonal- or polyclonal antibody, from which the precise epitope is not known. The Pro-C3 ELISA utilized a monoclonal antibody specific for the PIIINP cleavage site resulting in a stronger surrogate for collagen III formation. This has been described in Nielsen et al. [29]. In the present work we confirmed that PRO-C3 reflects ECM remodelling and liver fibrogenesis in HIV/HCV coinfected patients. Most importantly, PRO-C3 seems to be a valuable biomarker for liver fibrosis stage and portal hypertension in HIV/HCV coinfection.

Furthermore, systemically assessed type IV (C4M) and V collagen (C5M) fragments were significantly higher in HIV/HCV-co-infected patients with portal hypertension. Interestingly, the level of C4M and C5M reflects not only the release of ECM-fragments, but also the turnover of ECM [18], and thereby may mirror the extent and activity of fibrotic processes. In contrast to PRO-C3, C4M is a marker of basement membrane degradation [30]. But similarly to the formation marker PRO-C3, C4M levels were also shown to be elevated in BDL and CCl4-treated rats [18]. In addition, we showed recently that C4M together with PRO-C3 has the highest diagnostic power as single marker to identify clinically relevant HVPG in patients with alcoholic cirrhosis [17]. Thus the present data are in alignment. Moreover, in a rat model of liver fibrosis C4M was correlated with the effect of antifibrotic treatment, which has still not deep validated in human studies [18]. All these studies have demonstrated that collagen degradation occurs in fibrotic processes and is may act as a mirror of the synthesis of collagen at a similar extent as the synthesis of collagen. Once again this study reinforces the strong causal association between ECM remodeling and portal hypertension. The present study confirms the importance of PRO-C3 and C4M as predictor of portal hypertension also in patients with HIV/HCV coinfection.

Interestingly, this study underlines a potential role of collagen type V degradation marker in the HIV/HCV patients. Fibrosis of lymphoid tissue has been described as a milestone of disease progression in HIV, and besides spleen, gut and lymph nodes, the liver is a major organ involved in the immune response towards pathogens [39]. In the presence of additional HCV infection, the liver disease may be the major couse of morbidity and mortality and this study is the first to highlight the potential pathogenetic role of collagen type V degradation in the progression of fibrosis and development of portal hypertension. Thus, our previous results in patients with alcoholic cirrhosis [18] also apply in patients with HIV/HCV-co-infection. As previously described type V collagen is a key player in formation and assembly of other collagens. Therefore it is not surprising that also increased levels of C5M has been described in diseases with abnormally high collagen remodeling such as chronic obstructive pulmonary disease [40] and idiopathic pulmonary fibrosis [40] and ankylosing spondylitis [31].

### Limitations

Although these patients were well characterized the present study has several limitations. We examined in this study a very selective small group of patients, which might be a bias of the study. Besides that, the validity of the results is strengthened, since they are well in line with previously published findings of correlation of HVPG with FIB-4 score, transient elastography assessed by Fibroscan, platelet count and aminotransferases levels [41]–[44]. Therefore, our identified markers may serve as early marker of liver injury, fibrosis and portal hypertension.

In conclusion, this study demonstrates that liver fibrosis and liver injury might be reflected by PRO-C3-levels in HIV/HCV-co-infected patients. PRO-C3 correlated with HVPG and parameters of hepatic dysfunction. Furthermore higher levels of C4M and C5M were associated with degree of portal hypertension.

## Supporting Information

Figure S1 **Correlation of HVPG with FIB4-Score (A), fibroscan (B) and PLT (C).** HVPG correlates with FIB4-Score (r_s_ = 0.628; p = 7*10^−7^) (A) as well with fibrosacan (r_s_ = 0.621; p = 9*10^−5^) (B) and inversely with PLT (r_s_ = −0.366; p = 0.016) (C). Data are presented using Spearman coefficient r_s_ and p-values.
(TIFF)

Table S1 **Overview of technical specification of the novel ECM assays presented in this study.**
(DOC)
