# Supplementary figures and images for: PRO-C3-Levels in Patients with HIV/HCV-Co-Infection Reflect Fibrosis Stage and Degree of Portal Hypertension
Source: PLoS One. 2014 Sep 29;9(9):e108544. doi: 10.1371/journal.pone.0108544 (PMC4180447; doi:10.1371/journal.pone.0108544)

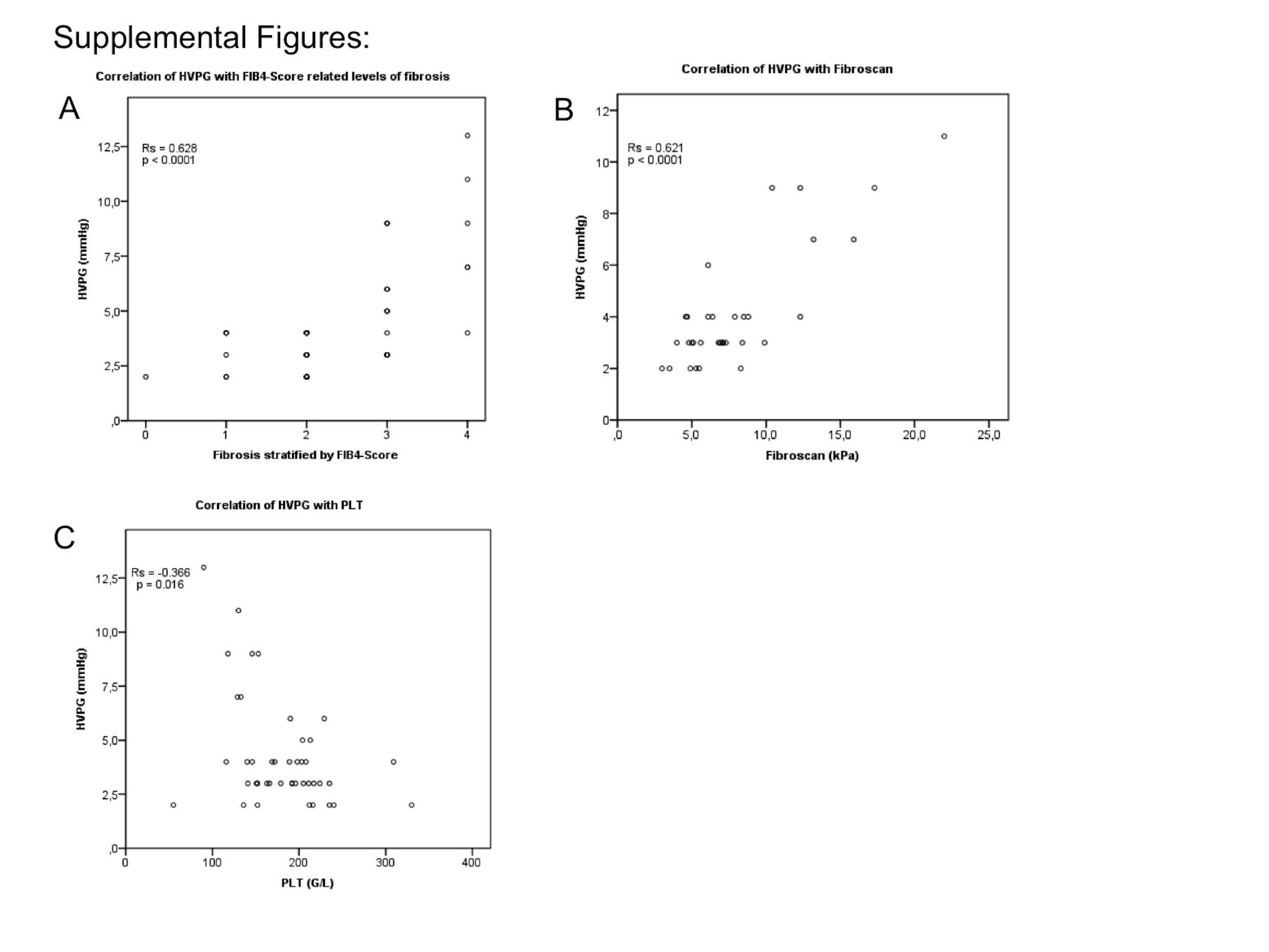

Supplement: Figure S1 — Correlation of HVPG with FIB4-Score (A), fibroscan (B) and PLT (C). HVPG correlates with FIB4-Score (rs = 0.628; p = 7*10−7) (A) as well with fibrosacan (rs = 0.621; p = 9*10−5) (B) and inversely with PLT (rs = −0.366; p = 0.016) (C). Data are presented using Spearman coefficient rs and p-values. (TIFF) [file pone.0108544.s001.tiff]
