# Supplementary material for: PRO-C3-Levels in Patients with HIV/HCV-Co-Infection Reflect Fibrosis Stage and Degree of Portal Hypertension
Source: PLoS One. 2014 Sep 29;9(9):e108544. doi: 10.1371/journal.pone.0108544 (PMC4180447; doi:10.1371/journal.pone.0108544)
Supplement: Table S1 — Overview of technical specification of the novel ECM assays presented in this study. (DOC) [file pone.0108544.s002.doc]

***Supporting Information Table S1:*** Overview of technical specification of the novel ECM assays presented in this study

| **Assay name** | **Target** | **Antibody type** | **Detectionrange (ng/mL)** | **Intra-assayvariation (%)** | **Inter-assayvariation (%)** |
| --- | --- | --- | --- | --- | --- |
| PRO-C3 | MMP degraded n-terminal | Monoclonal | 0.9 - 200 | 4.1 | 11 |
|  | propeptide of type III collagen |  |  |  |  |
| C4M | MMP-2/9 degraded | Monoclonal | 0.6 - 100 | 4.8 | 12.1 |
|  | type IV collagen |  |  |  |  |
| C5M | MMP-2/9 degraded | Monoclonal | 11.3 -1000 | 4.4 | 9.1 |
|  | type V collagen |  |  |  |  |
